# Impact of the COVID-19 pandemic on emergency and elective hip surgeries in Norway

**DOI:** 10.1080/17453674.2021.1898782

**Published:** 2021-03-24

**Authors:** Karin Magnusson, Jon Helgeland, Mari Grøsland, Kjetil Telle

**Affiliations:** a Norwegian Institute of Public Health, Cluster for Health Services Research , Oslo , Norway ;; b Lund University, Faculty of Medicine, Department of Clinical Sciences Lund, Orthopaedics, Clinical Epidemiology Unit , Lund , Sweden

## Abstract

Background and purpose — Many countries implemented strict lockdown policies to control the COVID-19 pandemic during March 2020. The impacts of lockdown policies on joint surgeries are unknown. Therefore, we assessed the effects of COVID-19 pandemic lockdown restrictions on the number of emergency and elective hip joint surgeries, and explored whether these procedures are more/less affected by lockdown restrictions than other hospital care.

Patients and methods — In 1,344,355 persons aged ≥ 35 years in the Norwegian emergency preparedness (BEREDT C19) register, we studied the daily number of persons having (1) emergency surgeries due to hip fractures, and (2) electively planned surgeries due to hip osteoarthritis before and after COVID-19 lockdown restrictions were implemented nationally on March 13, 2020, for different age and sex groups. Incidence rate ratios (IRR) reflect the after-lockdown number of surgeries divided by the before-lockdown number of surgeries.

Results — After-lockdown elective hip surgeries comprised one-third the number of before-lockdown (IRR ∼0.3), which is a greater drop than that seen in all-cause elective hospital care (IRR ∼0.6). Men aged 35–69 had half the number of emergency hip fracture surgeries (IRR ∼0.6), whereas women aged ≥ 70 had the same number of emergency hip fracture surgeries after lockdown (IRR ∼1). Only women aged 35–69 and men aged ≥ 70 had emergency hip fracture surgery rates after lockdown comparable to what may be expected based on analyses of all-cause acute care (IRR ∼0.80)

Interpretation — It is important to note for future pandemics management that lockdown restrictions may impact more on scheduled joint surgery than other scheduled hospital care. Lockdown may also impact the number of emergency joint surgeries for men aged ≥ 35 but not those for women aged ≥ 70.

Because of COVID-19, Norway implemented early one of the strictest lockdown policies of all countries. The lockdown measures are believed to have limited the spread of the virus in this country dramatically, but on the other hand, may have had several unknown negative side effects on the planned and acute care for vulnerable groups. As an example, people with osteoporotic fractures and osteoarthritis are often elderly and fragile, with a high need for care to prevent long-term disability and death. The conditions are often managed by the most commonly performed surgical joint procedure worldwide: total hip arthroplasty (Learmonth et al. 2007). Whereas acute hip fracture surgeries should be performed within 24 hours according to national guidelines (NOF 2018), surgeries due to hip osteoarthritis are typically planned weeks or months in advance (Zhang et al. 2008).

The impact of the COVID-19 lockdown restrictions on such typically acute and elective care for age-related conditions that require hospitalization is currently unknown, but can be hypothesized to be major, at least for elective care. Also, if there is an effect on acute care, knowledge of which population groups to a lesser extent need, or make use of, acute care is important in the future handling of pandemics. Such analyses may also provide knowledge for future natural experiments evaluating whether any care is in fact unnecessary in a long-term perspective (Moynihan et al. 2020). Thus, we assessed the effects of the COVID-19 pandemic lockdown restrictions on acute and elective inpatient care in Norway during spring 2020, using surgeries for hip fractures and surgeries for hip osteoarthritis as examples.

## Methods

We utilized data from the BEREDT C19 register, which is a newly developed emergency preparedness register aiming to provide rapid knowledge of the spread of the COVID-19 virus and how spread as well as measures to limit spread affect the population’s health, use of healthcare services, and health-related behaviors (Norwegian Institute of Public Health 2020). The register currently consists of electronic patient records from all hospitals in Norway (NPR), and data from the Norwegian Surveillance System for Communicable Diseases (MSIS) and the Norwegian Intensive Care and Pandemics Register (NIPaR), which are merged on the personal identification number and updated daily, with a range of other registry linkages currently ongoing. The register covers all data from hospitals (inpatient, outpatient, and day-care), with complete diagnostic and procedure codes from January 1, 2020 until the pandemic is over and has been evaluated. In the current study, our population included everyone in Norway registered with acute or elective inpatient care and we restricted our sample to the age groups to which diagnoses of hip osteoarthritis and fractures apply (age 35 or more).

### Outcomes

Besides studying all registered inpatient care coded with emergency grades acute vs. elective (any cause), we studied the number of patients hospitalized with the outcomes (1) emergency surgeries due to hip fractures, and (2) electively planned surgeries due to hip osteoarthritis. Hip fracture surgeries were identified as having ICD-10 codes S72* (main diagnosis or other diagnosis) in combination with NCSP procedure code NFJ* and/or NFB*, and an emergency grade coded as acute. Hip osteoarthritis surgeries were identified as having ICD-10 codes M16* (main diagnosis or other diagnosis) and procedure code NCSP NFB* and an emergency grade coded as elective.

### Statistics

We assessed the described outcomes prior to and after lockdown restrictions were implemented in Norway on March 13, 2020, i.e., in the period January to May 2020. We first studied all-cause acute and elective inpatient care using a Poisson regression model with the daily number of hospitalizations as outcome (i.e., acute and elective emergency grades in separate analyses) and time as explanatory variable (allowing for overdispersion in negative binomial models had negligible impacts on results). Thus, we categorized dates in 5 x 2-week periods before March 13, 2020, and 5 x 2-week periods after March 13, 2020, covering a total time period from January 3, 2020 to May 21, 2020. To observe trends in number of surgeries over time, we compared the incidence rate ratios (IRR) for all the 2-week periods with the base level, which was defined as the two first weeks of January, starting from January 3. We also compared IRR in the 10 weeks before and after lockdown restrictions were implemented nationally in Norway on March 13, 2020, using the period of 10 weeks before lockdown as base level (Jannuary 3–March 12). The IRR should be interpreted as the estimated number of surgeries for the given period divided by the estimated number of surgeries in the base level period.

We then repeated these analyses for emergency hip fracture surgeries and elective hip osteoarthritis surgeries. Also, to explore whether any hip surgery patient groups may be more affected by lockdown restrictions in terms of their healthcare use than would be expected from our analyses of all-cause acute and elective hospitalizations, we stratified the analyses on age (35–69 years vs. 70 and above), for men and women separately. Analyses were adjusted for weekends and holidays. Finally, we predicted number of unperformed surgeries in the period after March 13, 2020 from the same Poisson regression model, conditional on weekdays. We used Stata version 16.1 (StataCorp, College Station, TX, USA) for all analyses.

### Ethics, funding, and potential conflicts of interest

Institutional board review was conducted, and the Ethics Committee of South-East Norway confirmed (June 4, 2020, #153204) that external ethical board review was not required. The study was funded by the Norwegian Institute of Public Health. No external funding was received, and the authors declare no conflicts of interests.

## Results

**Figure 1.**
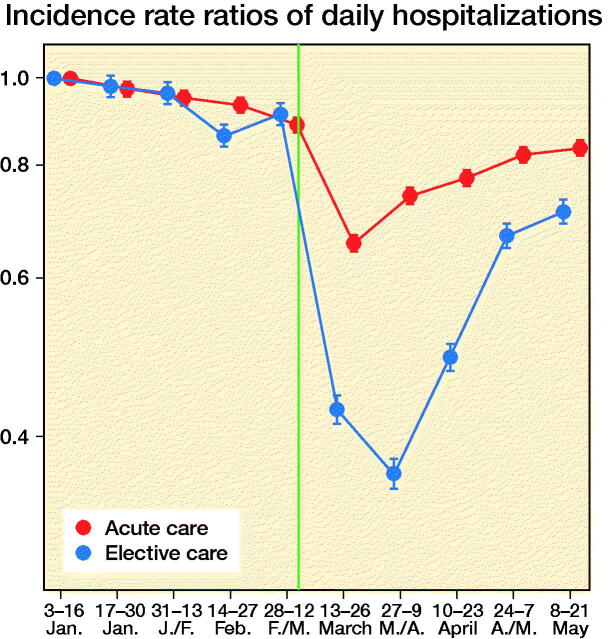
Incidence rate ratios of daily hospitalizations (any cause) with acute and elective emergency grade in Norway, January 3, 2020–May 21, 2020, with January 3–January 16 as base level, with 95% confidence intervals. Red dots/line = acute care. Blue dots/line = elective care. The red and blue 2-weekly dots are graphed next to each other for improved readability. Vertical line represents the national implementation of lockdown strategies on March 13, 2020.

BEREDT C19 comprised 1,344,355 individuals with at least one contact with specialist care from January 3 2020 to May 21 2020. Of those aged 35 or more having > 24-hour hospitalizations (inpatients), we observed 73,091 emergency and 187,714 elective hospitalizations (persons could be counted in both groups). Persons receiving elective inpatient care had half the rate of care after lockdown (n = 26,360 hospitalizations) compared with before lockdown (n = 46,731 hospitalizations) (IRR 0.56, 95% confidence interval [CI] 0.56–0.57 as compared with base levels before lockdown, IRR 1) (Figure 1). Also, acute care occurred at a 20% lower rate after lockdown (n = 83,838 hospitalizations) than before (n = 103,875 hospitalizations) (IRR 0.81, CI 0.80–0.81) (Figure 1).

We observed 2,701 new hip surgeries due to osteoarthritis and 3,650 new hip surgeries due to fractures throughout the study period, i.e., in persons without prior prosthesis surgery in any of the hip joints. Before March 13, 2020, the daily number of new hip surgeries corresponded to that reported for the same time period in previous years (Helse Bergen 2014). The rate of elective hip surgeries due to osteoarthritis dropped substantially after lockdown restrictions were implemented on March 13, 2020, with similar observations across age and sex groups (Figure 2). For men aged 35–69, there was also a slight decrease in the rate of emergency hip surgeries due to fractures (Figure 2).

Figure 2.Incidence rate ratios (y-axis, IRR) of emergency hip surgeries due to fracture (red) and the IRR of elective hip surgeries due to osteoarthritis (blue) in Norway, January 3, 2020–May 21, 2020 with January 3–January 16 as base level with 95% confidence intervals (CI). Red dots/line = emergency hip fracture surgeries. Blue dots/line = elective hip osteoarthritis surgeries. The red and blue 2-weekly dots are graphed next to each other for improved readability. Vertical line represents the national implementation of lockdown strategies on March 13, 2020.

**Figure:**
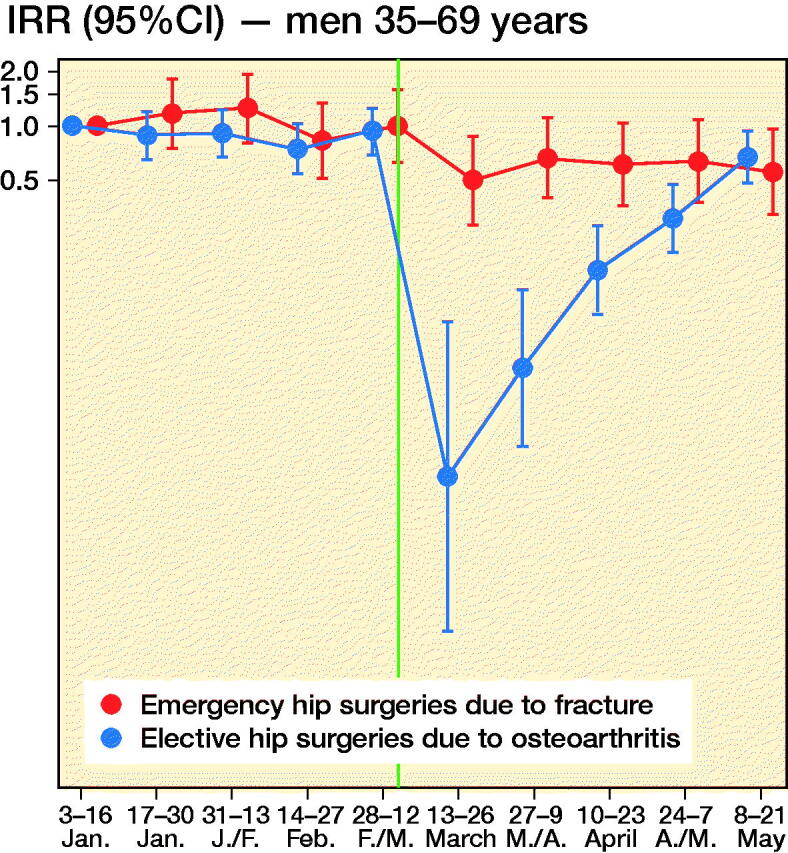

**Figure:**
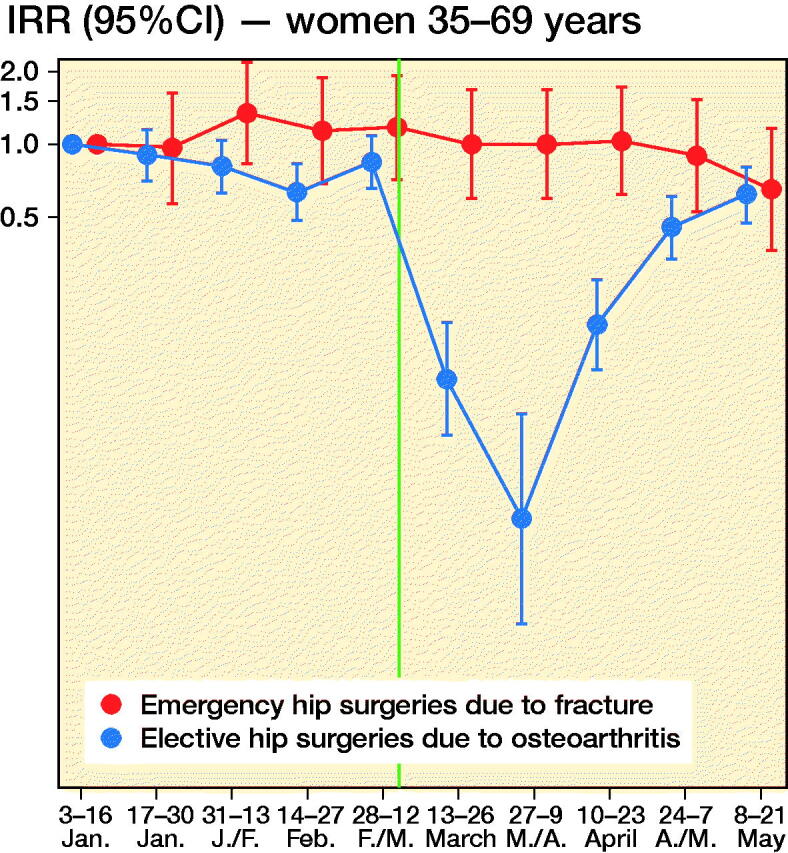

**Figure:**
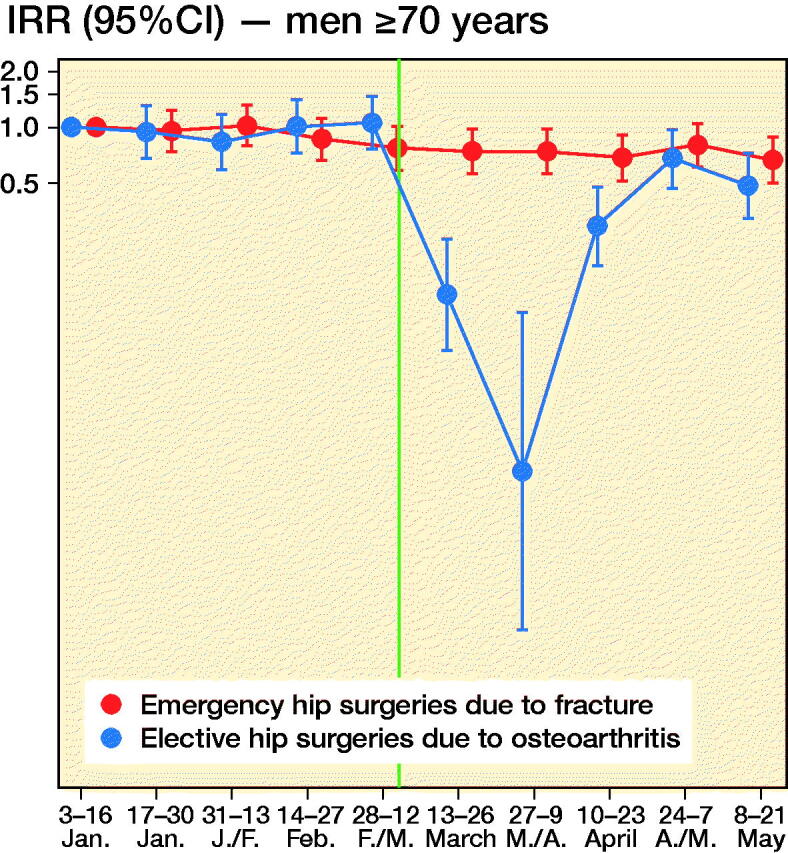

**Figure:**
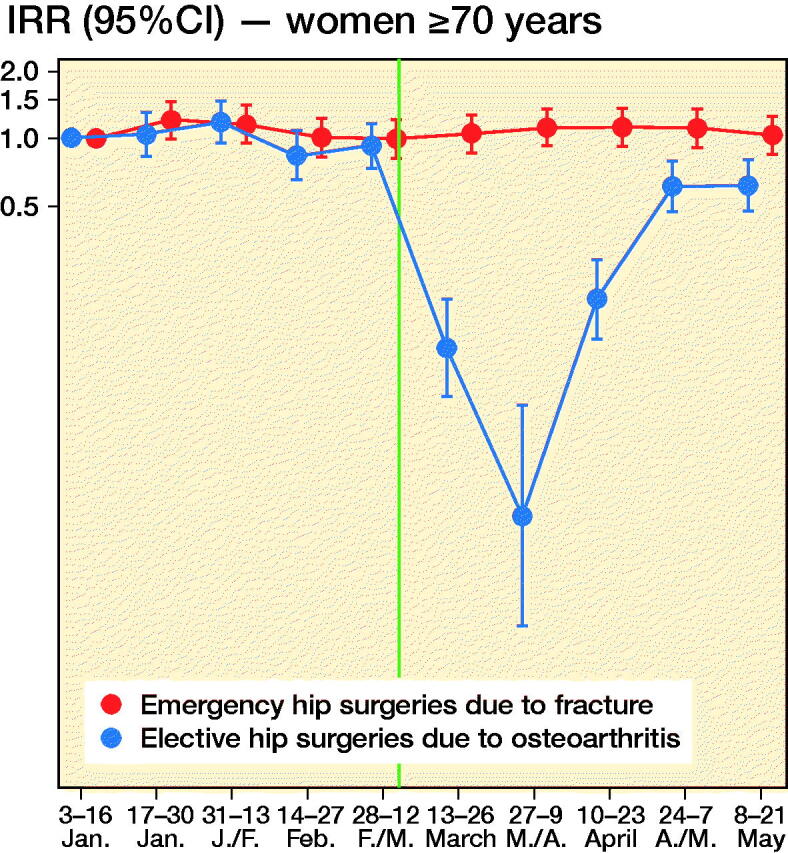

When compared with what may be expected, based on the average after-lockdown drop in all-cause elective and all-cause acute hospitalizations, we observed large deviations for our musculoskeletal outcomes. For elective hip osteoarthritis surgeries, IRRs were half that observed for elective all-cause hospitalization, for all age and sex strata (IRR ∼0.3 for elective hip osteoarthritis surgeries vs. IRR ∼0.6 for all-cause elective care) (Table). This would imply that around 1,400 planned hip surgeries in Norway have not been performed due to the COVID-19 pandemic and would need to be treated elsewhere or scheduled for surgery on another date (estimated number of unperformed hip osteoarthritis surgeries after March 13, 2020 = 1,417, CI 1,392–1,440).

For emergency hip fracture surgeries there were large variations by age and sex. Men aged 35–69 had a lower rate of emergency hip fracture surgery than of all-cause acute care (IRR ∼0.6 vs. ∼0.8), whereas women aged 70 or more had a higher such rate (IRR ∼0.8 vs. ∼1.0) (see Table). In contrast, women aged 35–69 and men aged ≥ 70 had hip fracture surgery rates comparable to what may be expected based on analyses of all-cause acute care (IRR ∼0.80) (see Table). Altogether, lockdown restrictions may have given a reduction of ∼200 acute events requiring immediate hip fracture surgery (estimated number of avoided hip fractures after March 13, 2020 = 210, CI 184–236). 

## Discussion

In this study based on data from the BEREDT C19—the Norwegian emergency preparedness register—we report a sudden and steep decrease in the daily number of planned hip joint surgeries, beginning on the day after lockdown restrictions were implemented in Norway on March 13, 2020. This decrease was greater than the decrease in other (all-cause) elective inpatient care for all age and sex groups. Interestingly, we also report a consistent decrease in all-cause acute inpatient care that was found only partly in age- and sex-specific analyses of emergency hip joint fracture surgeries; lockdown restrictions may impact on the number of acute joint surgeries for middle-aged and elderly men (aged ≥ 35) as well as for middle-aged women (age 35–69), but not for elderly women (aged ≥ 70).

The observed decrease of elective care including hip surgeries is not surprising, and sheds new light on a recent report on effects of lockdowns on elective care globally (COVIDSurg Collaborative, 2020). Here, we additionally show that the activity of elective surgeries was reduced more than other elective activities in inpatient care, and that it increased rather quickly again, as authorities gained control over the spread of the pandemic during April 2020. However, surgery rates were not back to normal (437 elective hip surgeries per 14-days periods during 2019 (Nasjonal kompetansetjeneste for leddproteser og hoftebrudd 2019)) by the end of May 2020 and around 1,400 elective hip osteoarthritis surgeries would have to be rescheduled or treated nonoperatively in primary care. Figure 2 shows that the activity recovered approximately equally for the different age and sex groups, although there may be minor variations.

For emergency hip surgeries due to fractures, we observed a somewhat unexpected decrease in incidence that was more evident for men than for women. Hospitals were not instructed to limit access to acute care, so the observed decrease may be explained by the fact that people stayed at home/inside more, which reduced the risk of falls and subsequent hip fractures. If so, the non-decreasing incidence of hip fractures in women may be explained by female hip fractures more frequently being a result of intrinsic causes like bone mineral density (Emaus et al. 2009). Whereas all age and sex groups had fewer hip fracture surgeries after lockdown, women aged ≥ 70 had a slightly increased hip fracture surgery rate, with an additional 4–25 surgeries occurring in the 10 weeks after lockdown compared with the 10 weeks before lockdown.

Our findings may have implications for the handling of future new outbreaks of COVID-19. First, our data show that when elective healthcare and other parts of society are locked down by the authorities, the instructions are followed by the hospitals and the use of elective care decreases to a similar magnitude for men and women, young and elderly. Using hip osteoarthritis surgery as an example, we also show that elective joint surgery rates decrease more than other elective inpatient care after lockdown restrictions are implemented. Second, the lockdown restrictions likely also impacted on the need for/use of acute health are, and did so to a different extent for men and women when exemplified using emergency hip fracture surgeries. Thus, our study implies that when policymakers consider lockdown of certain elective hospital activities during future outbreaks, it might affect people with musculoskeletal pain, and in particular men, disproportionally severely. Considering that chronic pain may lead to permanent work disability, policymakers may want to be more careful in locking down healthcare services for this large group of persons in the future. We suggest the future effects of unperformed hip surgeries due to lockdown as a topic for future study. Also, our findings suggest that different population groups have different levels of anxiety in seeking healthcare when a pandemic is present, i.e., people may be afraid of seeking healthcare because of risk of infection. We suggest future studies to further explore the causes for the different reductions in acute hip surgeries in elderly and young men and women.

Some important limitations should be mentioned. First, we could not study whether the effect of lockdown restrictions is causal. For example, it is possible that the decrease in emergency hip fracture surgeries in men aged 35–69 is partly due to seasonal variations. However, we note that the number of surgeries prior to March 13, 2020 was similar to that reported for previous years (Helse Bergen 2014). Also, our findings apply only to Norwegian conditions and countries with similar healthcare services, healthcare organization, and demography to Norway. Future studies should explore the effects of lockdown restrictions on healthcare use comparing different countries’ lockdown strategies. A second limitation may be that we could not distinguish between experiencing joint pain and/or an acute event and seeking healthcare. Thus, as described above, there may be age and sex differences in care-seeking behavior that we could not account for here. Finally, there may be several potential competing risks in our sample. For example, persons hospitalized for cancer treatment may be unlikely to experience a hip fracture because they are indoors more. However, our goal was not to study disease etiology; rather, we give an overview of potential impacts individuals in need of hip joint surgery may experience as a result of lockdown restrictions.

In conclusion, we show that the lockdown restrictions implemented in Norway due to the spread of the COVID-19 pandemic reduced the use of elective inpatient care, but also acute inpatient care. In particular, we report that men and midlife age groups had a lower rate of emergency hip fracture surgeries after than before lockdown. We believe it is important to report these findings for improved knowledge, allowing for optimal management of future pandemics on a similar or larger scale.

The authors would like to thank the Norwegian Directorate of Health, in particular Director for Health Registries Olav Isak Sjøflot and his department, for excellent cooperation in establishing the emergency preparedness register. They would also like to thank Gutorm Høgåsen, Anja Lindman, and Ragnhild Tønnessen for their invaluable efforts in the work on the register. The interpretation and reporting of the data are the sole responsibility of the authors, and no endorsement by the register is intended or should be inferred. The authors would also like to thank everyone at the Norwegian Institute of Public Health who has been part of the outbreak investigation and response team.

**Table 1.** Hip surgeries 10 weeks after compared with 10 weeks before (base level) the implementation of national lockdown on March 13, 2020. All events represent > 24-hour hospitalizations

	Men	Men	Women	Women
Factor	35–69 years	≥ 70 years	35–69 years	≥ 70 years
All-cause elective care				
IRR (CI) after lockdown vs. before	0.58 (0.58–0.58)	0.60 (0.60–0.60)	0.55 (0.54–0.55)	0.53 (0.52–0.53)
Elective hip surgery (osteoarthritis)				
No. of surgeries before lockdown	394	349	580	736
No. of surgeries after lockdown	105	115	193	229
IRR (CI) after lockdown vs. before	0.27 (0.26–0.27)	0.33 (0.32–0.34)	0.33 (0.33–0.34)	0.31 (0.31–0.32)
Estimated no. (CI) of cancelled surgeries				
after lockdown vs. before	289 (284–294)	234 (229–239)	387 (380–393)	507 (499–514)
All-cause acute care				
IRR (CI) after lockdown vs. before	0.83 (0.83–0.83)	0.81 (0.81–0.81)	0.79 (0.79–0.80)	0.79 (0.79–0.80)
Emergency hip surgery (fractures)				
No. of surgeries before lockdown	189	539	163	1,039
No. of surgeries after lockdown	107	426	133	1,054
IRR (CI) after lockdown vs. before	0.56 (0.55–0.58)	0.79 (0.79–0.80)	0.82 (0.79–0.84)	1.01 (1.00–1.02)
Estimated no. (CI) of avoided hip				
fractures after lockdown vs. before	82 (78–86)	113 (106–120)	30 (25–34)	15 (4–25) ^a^

**^a^**Estimated increase.

CI: 95% confidence interval. IRR: incidence rate ratio.
